# Stress ulcer prophylaxis in adult neurocritical care patients—no firm evidence for benefit or harm

**DOI:** 10.1186/s13054-016-1188-6

**Published:** 2016-02-05

**Authors:** Mette Krag, Anders Perner, Jørn Wetterslev, Morten Hylander Møller

**Affiliations:** 1 Department of Intensive Care 4131, Copenhagen University Hospital Rigshospitalet, Blegdamsvej 9, 2100 Copenhagen, Denmark; 2 Copenhagen Trial Unit, Centre for Clinical Intervention Research, Copenhagen University Hospital Rigshospitalet, Blegdamsvej 9, 2100 Copenhagen, Denmark

In volume 19 (2015) of *Critical Care*, Liu et al. [1] present a systematic review of risks and benefits of stress ulcer prophylaxis (SUP) in adult neurocritical care patients. A total of eight randomised controlled trials (RCTs) on SUP with proton pump inhibitors or histamine-2 receptor antagonists versus placebo or no prophylaxis in neurocritical care patients was assessed. The authors conclude that SUP is superior to placebo/no prophylaxis in reducing gastrointestinal (GI) bleeding and all-cause mortality, while not increasing the risk of nosocomial pneumonia [1].

We would like to thank the authors for highlighting an important topic with significant clinical equipoise; however, we are worried that biased results and conclusions are presented. First, the review holds methodological limitations, including the fact that no predefined sensitivity analysis with continuity correction in the zero-event trials was planned or performed, and importantly the risk of random errors using trial sequential analysis (TSA) was not assessed. Applying TSA to the three trials with lowest risk of bias [1] suggests that an estimated required information size of 2005 patients and 1790 patients are needed to confirm or reject a 20 % relative risk reduction or increase in GI bleeding and all-cause mortality, respectively. Consequently, the cumulative meta-analysis presented, including 829 patients, is severely underpowered, with a high risk of presenting spurious findings [2]. Second, all included trials had a high risk of bias, which increases the risk of overestimating the benefit and underestimating harm [3]. Third, the authors have not assessed the potential harms of SUP adequately, as no data were included on the important patient-centred outcome measures of *Clostridium difficile* infection and cardiovascular events [4]. Finally, applying Grading of Recommendations Assessment, Development and Evaluation (GRADE) [5] to the results highlights that the quantity and quality of evidence are low (downgraded for risk of bias and imprecision), and that there is no firm evidence for benefit or harm of SUP versus placebo/no prophylaxis in adult neurocritical care patients.

In summary, adequately powered, high-quality RCTs are needed to inform us on whether adult critically ill patients, including those receiving neurocritical care, benefit from routine treatment with SUP.

## Abbreviations

GI: gastrointestinal
GRADE: Grading of Recommendations Assessment, Development and Evaluation
RCT: randomised controlled trial
SUP: stress ulcer prophylaxis
TSA: trial sequential analysis
