# Supplementary material for: Stress ulcer prophylaxis in adult neurocritical care patients—no firm evidence for benefit or harm
Source: Crit Care. 2016 Feb 5;20:22. doi: 10.1186/s13054-016-1188-6 (PMC4743209; doi:10.1186/s13054-016-1188-6)
Supplement: Related texts

# Reply: Authors’ response

**Authors:** Bolin Liu, Shujuan Liu, Anan Yin, Javed Siddiqi

We appreciate the attention and correspondence from Krag et al. First, as they pointed out, TSA might be a new promising attempt to reduce the random error risk and to increase the information size of a cumulative meta-analysis. We believed that the application of this analysis may add more statistical confidences to the conclusion. However, the present meta-analysis was performed in compliance with the recommendations of the Cochrane Collaboration for intervention reviews [6]. At present, it is not compulsory to conduct TSA in a meta-analysis. Second, zero-cell corrections are necessary for the Mantel–Haenszel methods if the same cell is zero in all included studies [7], which was not the case in the present study. Finally, and most importantly, in line with our conclusion, Krag et al. are conservative with the superiority of SUP versus placebo/no prophylaxis in adult neurocritical care patients. As indicated in our meta-analysis, the robustness of our conclusions is limited by the lack of trials with low risk of bias, sparse data, heterogeneity among trials, and concern regarding small trial bias [1]. While the present meta-analysis favoured the use of SUP in adult neurocritical care patients based on the preliminary evidence to date, more definitive conclusions can only be drawn from larger, well-designed, RCTs.
